# QSIRS Can Improve Accuracy of QSOFA and SIRS in Prediction of Mortality in Surgical Emergencies

**DOI:** 10.1055/s-0041-1733831

**Published:** 2021-08-03

**Authors:** Abdourahmane Ndong, Adja Coumba Diallo, Jacques Noel Tendeng, Amadou Ibra Diallo, Mohamed Lamine Diao, Sylvain Assega Sagna, Saer Diop, Diago Anta Dia, Daouda Diouf, Bayo Ismael Racine, Philippe Manyacka Ma Nyemb, Ibrahima Konaté

**Affiliations:** 1 Department of Surgery, Gaston Berger University, Saint-Louis, Senegal; 2 Department of Public Health and Preventive Medicine, Cheikh Anta Diop University, Dakar, Senegal

**Keywords:** surgery, emergency, score, QSOFA, SIRS, QSIRS

## Abstract

**Background** The quick sequential organ failure assessment (QSOFA) score and the systemic inflammatory response syndrome (SIRS) criteria were developed to predict the risk of sepsis and death in patients received in emergency. To improve sensitivity in predicting death, the association of the two scores was proposed under the term QSIRS (QSOFA + SIRS). Our aim was to determine the accuracy of QSOFA, SIRS, and QSIRS in prediction of mortality in surgical emergencies, and to compare these scores.

**Patients and Methods** This is a prospective study over a period of 1 year. Patients older than 15 years who presented a digestive surgical emergency (bowel obstruction, peritonitis, appendicitis, strangulated hernia) were included. For each score, the specificity, the sensitivity, the positive predictive value, the negative predictive value, and areas under the receiver operating characteristic (ROC) curve (AUC) were compared.

**Results** One hundred and eighteen patients were included and 11 deaths were recorded (9.3%). There was a statistically significant relationship between each score and death (QSOFA *p* = 0.01, SIRS *p* = 0.003, and QSIRS *p* = 0.004). The realization of the ROC curve found a higher AUC for QSIRS (0.845 [0.767–0.905]) compared with QSOFA (0.783 [0.698–0.854]) and SIRS (0.737 [0.648–0.813]). QSIRS (90.9%) had a higher sensitivity compared with the two other scores alone (SIRS = 81.9% and QSOFA = 36.3%).

**Conclusion** Our study found that QSIRS improves the ability to predict death in digestive surgical emergencies.

Surgical emergencies remain frequent in sub-Saharan Africa where they represent an important disease burden. In fact, 70% of deaths related to surgical emergencies occur in low-income countries. 1 Improving this prognosis requires early recognition of patients at high risk of death for optimal management. In this perspective, the prognostic scores constitute helpful decisional tool by objectively categorizing the risk of morbidity and mortality in patients. 2 The quick sequential organ failure assessment (QSOFA) score and the systemic inflammatory response syndrome (SIRS) criteria were initially developed to predict the risk of sepsis and therefore death in patients received in emergency. 3 To improve sensitivity in predicting death, the association of the two scores was proposed under the term QSIRS (QSOFA + SIRS). 4 5 Our aim was to determine the accuracy of QSOFA, SIRS, and QSIRS in prediction of mortality in surgical emergencies, and to compare these three scores.

## Patients and Methods

Our study was conducted at the regional hospital center of Saint-Louis. It is a prospective, descriptive, and analytical study. This is a study over a period of 1 year (May 1, 2017,–April 30, 2018). Patients older than 15 years who presented a digestive surgical emergency (bowel obstruction, peritonitis, appendicitis, and strangulated hernia) were included. Gynecological and traumatic etiologies were excluded. We studied age, sex, consultation delay, American Society of Anesthesiologists status, final diagnosis, and death within 30 days.

Blood pressure, temperature, heart rate, respiratory rate, Glasgow score, and the white blood cell (WBC) count were used to calculate the SIRS and QSOFA scores for every patient at admission.

- The QSOFA score is positive in the presence of two of the three following elements: systolic blood pressure ≤ 100 mm Hg, respiratory rate ≥ 22 breaths/min, and Glasgow score ≤ 14. 3
- SIRS was positive in the presence of two of the following four elements: temperature > 38.3 or < 36, heart rate > 90 beats/min, respiratory rate > 20 breaths/min, and WBC count > 12,000 or < 4,000/mm ^3^ . 3
- QSIRS score (as described by Green et al), was generated by combining the SIRS and the QSOFA scores for each patient. As both scores include a respiratory rate, only one respiratory rate was included in the final score, to avoid duplication. A cutoff of more than or equal to 20 breaths/min was used. A QSIRS score ≥ 2 was considered to be positive. 4

## Statistical Analysis

The qualitative variables were described in number with their proportion, and the quantitative variables as mean with their standard deviation. A univariate analysis between death and the different scores was performed. Pearson's chi-square test or Fischer's test was used with a difference considered significant when the *p* < 0.05. The odds ratio (OR) surrounded by its confidence interval (CI) measured the strength of the link.

For each score, the specificity, the sensitivity, the positive predictive value (PPV), and the negative predictive value (NPV) were calculated by the usual formulas. The ability to predict the death of the three scores was determined by receiver operating characteristic (ROC) curves and the different areas under the curve (AUC) with their CIs were compared. Statistical analysis was done on Medcalc 14.8.1.

## Results

One hundred and eighteen patients were included. The average age of the patients was 35.8 years with a minimum age of 16 and maximum age of 90 years. The standard deviation was 18.07. There were 94 men (79.6%) and 24 women (20.4%) (sex ratio of 3.9).

In 30 days of follow-up, 11 deaths were recorded (9.3%). Patient baseline characteristics are shown in Table 1 .

**Table 1:** Baseline characteristics of patients with surgical emergencies ( *n* = 118)

Variable		Number ( *n* )	Percentage (%)
Age (y)	< 50	92	78
	≥ 50	26	22
Gender	Male	94	79.6
	Female	24	20.4
ASA	I	95	80.5
	II	17	14.4
	III	6	5.1
Consultation delay (h)	< 48	32	27
	≥ 48	86	73
Diagnosis	Bowel obstruction	51	43.2
	Peritonitis	33	28
	Appendicitis	23	19.5
	Strangulated hernia	11	9.3
Temperature > 38.3 or < 36	Yes	43	36.4
	No	75	63.6
Systolic blood pressure ≤ 100 mm Hg	Yes	22	18.6
	No	96	81.4
Hearth > 90 beats/min	Yes	79	65.5
	No	39	34.5
Respiratory rate ≥ 20 breaths/min	Yes	66	55.9
	No	52	44.1
Glasgow score ≤ 14	Yes	100	84.7
	No	18	15.3
WBC count> 12,000 or < 4,000	Yes	67	56.8
	No	51	43.2
QSOFA	Positive	13	11.1
	Negative	105	88.9
SIRS	Positive	74	62.7
	Negative	44	37.3
QSIRS	Positive	59	50
	Negative	59	50
Death	Yes	11	9.3
	No	107	90.7

Abbreviations: ASA, American Society of Anesthesiologists; QSIRS, QSOFA + SIRS; QSOFA, quick sequential organ failure assessment; SIRS, systemic inflammatory response syndrome; WBC, white blood cell.

**Fig. 1:**
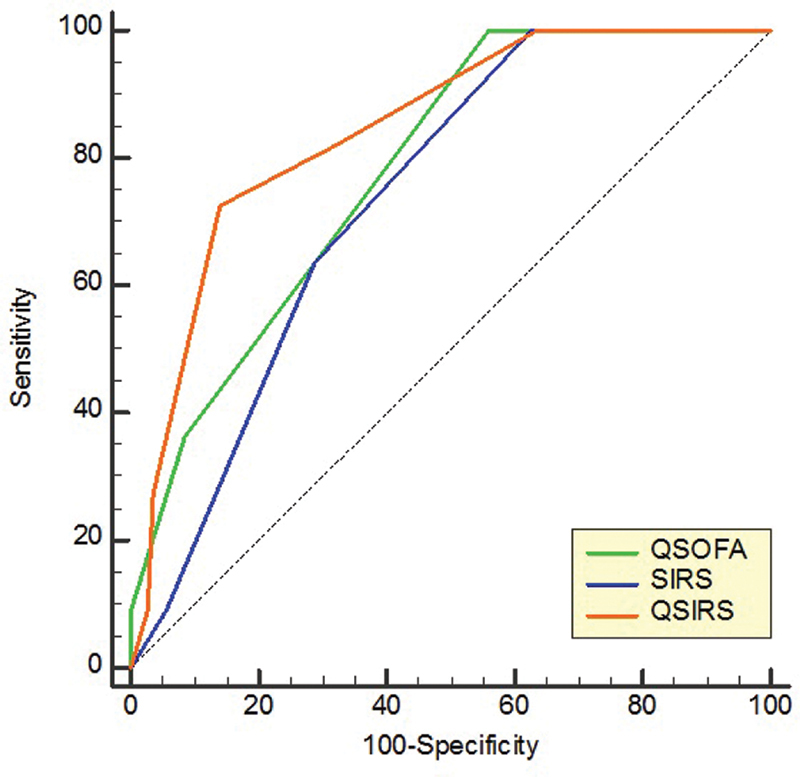
ROC curve between the different scores and mortality. QSOFA: AUC = 0.783 (0.698–0.854). SIRS: AUC = 0.737 (0.648–0.813). QSIRS: AUC = 0.845 (0.767–0.905). AUC, area under the curve; QSIRS, QSOFA + SIRS; QSOFA, quick sequential organ failure assessment; ROC, receiver operating characteristic; SIRS, systemic inflammatory response syndrome.

There was a statistically significant relationship between each score and death (QSOFA *p* = 0.01, SIRS *p* = 0.003, and QSIRS *p* = 0.004) ( Table 2 ). The realization of the ROC curve found a higher AUC for QSIRS (0.845 [0.767–0.905]) compared with QSOFA (0.783 [0.698–0.854]), and SIRS (0.737 [0.648–0.813]) ( Fig. 1 ).

**Table 2:** Univariate analysis between QSOFA, SIRS, QSIRS, and death ( *n* = 118)

Scores		Death		OR (CI)	*p* -Value
		Yes	No		
QSOFA	Positive	4	9	6.2 (1.5–25.3)	0.01
	Negative	7	98		
SIRS	Positive	9	38	8 (1.8–56.9)	0.003
	Negative	2	69		
QSIRS	Positive	10	49	11.8 (1.4–95.7)	0.004
	Negative	1	58		

Abbreviations: CI, confidence interval; OR, odds ratio; QSIRS, QSOFA + SIRS; QSOFA, quick sequential organ failure assessment; SIRS, systemic inflammatory response syndrome.

Sensitivity, specificity, PPV, and NPV are shown in Table 3 . QSIRS (90.9%) had a higher sensitivity compared with the two other score alone (SIRS = 81.9% and QSOFA = 36.3%).

**Table 3:** Sensitivity, specificity, PPV, and NPV of QSOFA, SIRS, and QSIRS

Score	Sensitivity (%)	Specificity (%)	PPV (%)	NPV (%)
QSOFA	36.3	91.5	30.7	93.3
SIRS	81.8	64.4	19.1	97.1
QSIRS	90.9	54.2	16.9	98.3

Abbreviations: NPV, negative predictive value; PPV, positive predictive value; QSIRS, QSOFA + SIRS; QSOFA, quick sequential organ failure assessment; SIRS, systemic inflammatory response syndrome.

## Discussion

This research was realized on patients presenting nontraumatic digestive surgical emergencies at the Saint-Louis hospital (Senegal). There are various etiologies of surgical emergencies, and due to the diverse physiological imbalances, that can occur, the evaluation of this type of patient is a challenge. 2 This explains the key place that hold the different scores in diagnostic and therapeutic management.

Our aim was to determine the accuracy of QSOFA, SIRS, and QSIRS in prediction of mortality in surgical emergencies. In clinical practice, sensitivity remains the most important criterion in predicting death. We found that QSIRS improves the sensitivity of QSOFA and SIRS. QSIRS had a higher sensitivity (90.9%) compared with the two other score (SIRS = 81.9% and QSOFA = 36.3%). Besides, QSIRS had also a higher AUC (0.845) in the ROC curve compared with QSOFA (0.783) and SIRS (0.737) taken alone.

The SOFA score was first developed to determine the existence of organ dysfunction in patients with sepsis. 6 It is a very high-performance score, but its main limitation is the need to perform biological tests which are not always available in all contexts. In fact, it requires the platelet count, bilirubinemia, and creatininemia. This can therefore lead to late identification of serious patients in clinical practice. 7 In this context, to facilitate the identification of patients potentially at risk of dying from sepsis, the QSOFA) was developed to recognize more easily these patients at risk. 3 8 The QSOFA score is very useful in low-resource setting because it does not require any paraclinical test and is feasible at the patient's bed. 4 This score has good sensitivity (96%) and specificity (87%) in predicting death in patients with sepsis. 8 The QSOFA allows rapid stratification of the risk of septic patients requiring an extended stay in intensive care at the same time as death in hospital.

The SIRS was introduced in 1991 for the rapid identification of sepsis, in the current era where various models for predicting complex clinical outcomes exist. 4 It is a very accessible because its achievement requires only clinical elements and the WBC count. However, SIRS is not necessarily associated with life-threatening organ dysfunction. Its sensitivity is high and it is present in many hospital patients, including those who do not have infection or death (low specificity). 3

For an improvement of the QSOFA and SIRS accuracy in prediction of morbidity and mortality, some authors have proposed the association of the two scores. 5

The first and only study to our knowledge that have evaluated this association is from Green et al that named this score QSIRS. By comparing QSIRS with other scores, including QSOFA and SIRS, they found that QSIRS had the highest accuracy in predicting mortality with an AUC of 0.731 (0.68–0.78). 4

Indeed, the combination of the two scores taking into account the clinical items and the WBC count improves the ability to predict sepsis and therefore death. Leukocytosis alone is an independent indicator of mortality and sepsis. 9 Early recognition of sepsis allows for adequate treatment (antibiotherapy, hydroelectrolytic resuscitation, and surgery).

## Limits

The small size of our cohort ( *n* = 118) is the main limitation of our study. In addition, this study was conducted in one hospital. Consequently, mortality data cannot be relatively transposed to other contexts. The limited number of events (11 deaths) can also influence the values of sensitivity and specificity. This is why the OR and AUC had wide CIs. In addition, sensitivity comparison was used in our study but do not fit with all situation. Even if QSIRS could help prioritize postoperative admission in intensive care unit to avoid complications, it's external validity should be studied more in further studies with adequate sample size.

## Conclusion

Our study found that QSIRS, which is the combination of QSOFA and SIRS, improves the ability to predict death in digestive surgical emergencies. More studies with larger cohorts are necessary to evaluate more deeply this new tool to determine its real value and its limits in clinical practice.
